# Ventriculoperitoneal shunt malfunction due to chronic cholecystitis: A case report

**DOI:** 10.1097/MD.0000000000020565

**Published:** 2020-06-19

**Authors:** Qi Yu, Chengjian Lou, Tianda Feng, Yunhui Liu

**Affiliations:** a Department of Neurosurgery, Shengjing Hospital of China Medical University; b Liaoning Clinical Medical Research Center in Nervous System Disease; c Liaoning Key Laboratory of Neuro-Oncology, Shenyang, China.

**Keywords:** chronic cholecystitis, laparoscopic cholecystectomy, normal pressure hydrocephalus, shunt malfunction, ventriculoperitoneal shunt

## Abstract

**Rationale:**

Ventriculoperitoneal shunt (VPS) is the most common treatment for idiopathic normal pressure hydrocephalus, a subtype of hydrocephalus characterized by gait disturbance, dementia, and urinary incontinence. However, while the malfunction of VPS is reported at a high rate, the involvement of chronic cholecystitis in shunt malfunction is rare.

**Patient concerns:**

A 73-year-old woman with idiopathic normal pressure hydrocephalus who received a VPS but subsequently developed chronic cholecystitis. The patient suffered from drowsiness and was unable to walk. Her family found that she presented with poor appetite and was bloated.

**Diagnoses:**

Chronic cholecystitis was confirmed through abdominal computed tomography, which showed a swollen, and enlarged gallbladder, and flatulence. A head computed tomography scan indicated hydrocephalus with enlarged ventricular system and paraventricular edema.

**Interventions:**

Laparoscopic cholecystectomy was performed successfully, requiring no further shunt manipulation.

**Outcomes:**

The patient's memory and cognitive ability were slightly impaired without a positive sign in the abdomen. No catheter or abdominal infection signs were observed during the following 3 months of follow-up.

**Conclusion:**

To the best of our knowledge, this report is the first to reveal that shunt malfunction may result from chronic cholecystitis, which induced the presently observed intra-abdominal hypertension.

## 1 Introduction

With an incidence of 181.7 per 100,000, more than 50% of which occur among adults older than 70 years,^[1]^ normal pressure hydrocephalus (NPH) can be categorized dichotomously: idiopathic and secondary. Patients with idiopathic NPH (iNPH) suffer from a combination of symptoms referred to as Hakim's triad: gait disturbance, urinary incontinence, and cognitive decline^[2]^; this last symptom first manifests as short-term memory disorder that eventually leads to dementia.^[3]^ In addition to the main symptoms, some patients also experience headaches and vertigo possibly consequent of the enlargement of the ventricles, whose registration on cerebral imaging is mandatory for the diagnosis iNPH.^[4]^ Although ventriculoperitoneal shunt (VPS) is the most widely accepted surgical treatment for iNPH, and the post-VPS improvement rates are reportedly high,^[5]^ some studies have associated the surgical intervention with potentially significant complication rates: Hebb and Cusimano reported a pooled mean shunt complication rate of as high as 38% (range 5%-100%), with a 22% (range 0%-47%) risk of additional surgery and 6% (range 0%-35%) combined rate of permanent neurological deficit and death.^[6]^ Among these complications, the most common is shunt malfunction. We herein report the rare case of an older patient with iNPH who developed chronic cholecystitis after receiving a shunt, which lead to the consequent failure of the VPS intervention.

## 2 Case report

The patient has provided informed consent for publication of the case, and the study design was approved by the Shengjing Hospital of China Medical University Institutional Review Board (IRB No.1698342)

**Figure 1:**
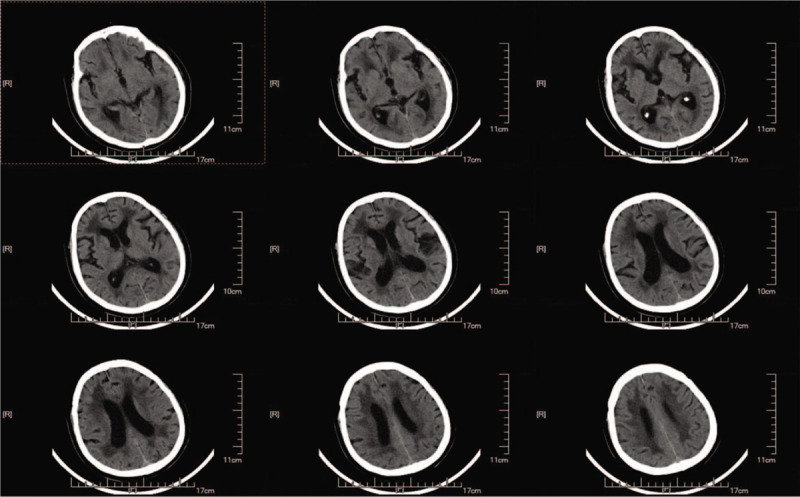
Preoperative head computed tomography scan obtained during first admission indicated ventricular enlargement and obvious paraventricular edema.

**Figure 2:**
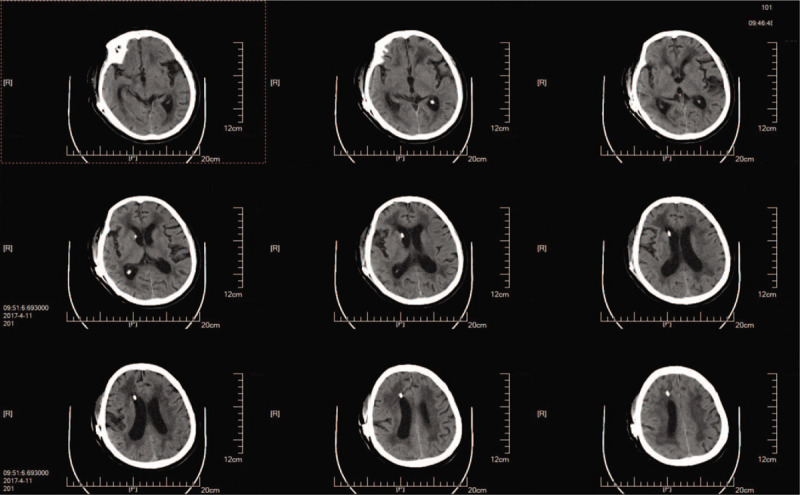
Postoperative head computed tomography scan obtained during first admission showed the relief of ventricular enlargement and paraventricular edema.

**Figure 3:**
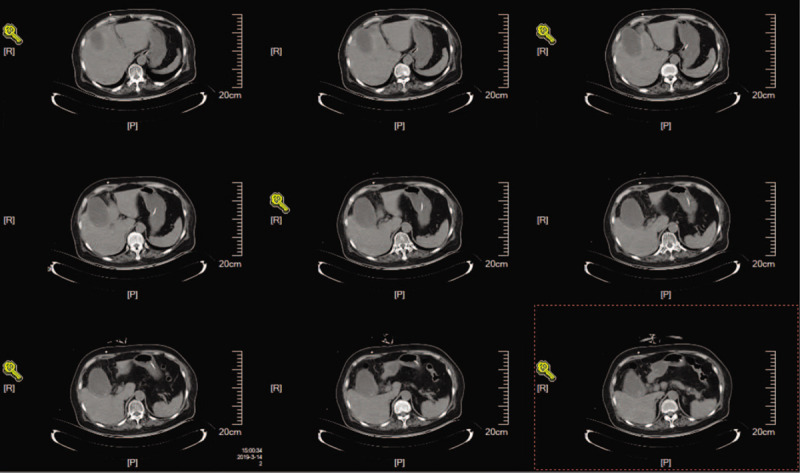
Preoperative abdominal computed tomography scan obtained during the second admission showed a swollen and enlarged gallbladder and flatulence.

**Figure 4:**
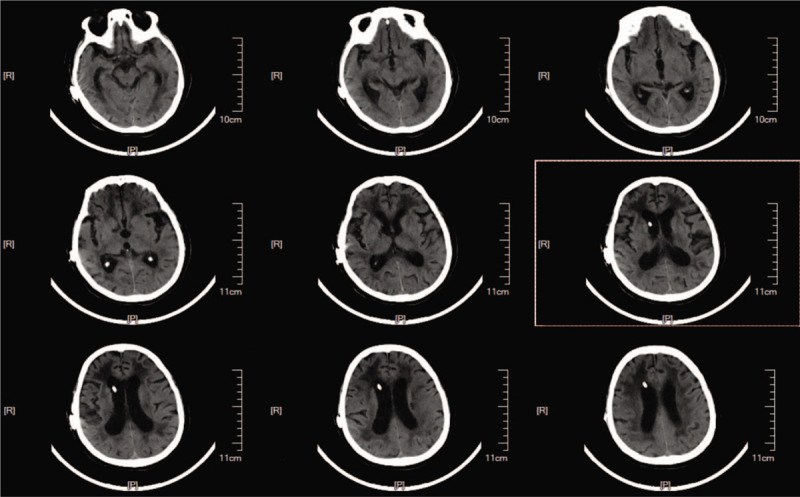
Preoperative head computed tomography scan obtained during the second admission indicated ventricular enlargement and the recurrence of obvious paraventricular edema.

A 73-year-old woman was diagnosed with iNPH after presenting with memory impairment and gait disturbance for more than 1 year. A head computed tomography (CT) scan indicated ventricular enlargement and obvious paraventricular edema (Fig. 1). The Tap test was positive, and she was indicated to receive a VPS using a Braun shunting system (proGAV + gravitational unit SA) (Fig. 2). With the operating pressure set at 2 cmH_2_O, she achieved clinical improvement within 1.5 years. Two months before her second admission, the patient suffered from drowsiness and was unable to walk. Her family found that she presented with poor appetite and was bloated. Chronic cholecystitis was confirmed through abdominal CT, which showed flatulence and a swollen, enlarged gallbladder (Fig. 3). After anti-inflammatory treatment, the patient's condition was stable, but her state of consciousness did not improve. A physical examination performed at the second admission revealed that she was in deep coma, was unresponsive to pain stimuli, and was unable to move her extremities. Abdominal distension was remarkable without tenderness. Relative to the CT findings obtained upon her discharge, a head CT scan indicated an enlarged ventricular system and paraventricular edema (Fig. 4). While these findings seemed to indicate that the opening pressure of the shunt did not match the intracranial pressure (ICP), the condition of our patient did not improve even after the operating pressure was decreased to 0 mmH_2_O.

**Figure 5:**
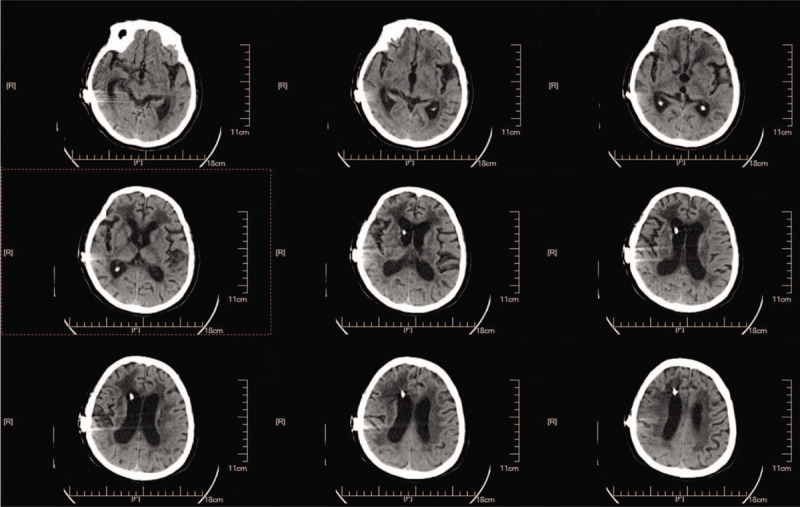
Preoperative abdominal X-ray scan performed during the second admission revealed the shunt tube to have been severely curled around the gallbladder.

**Figure 6:**
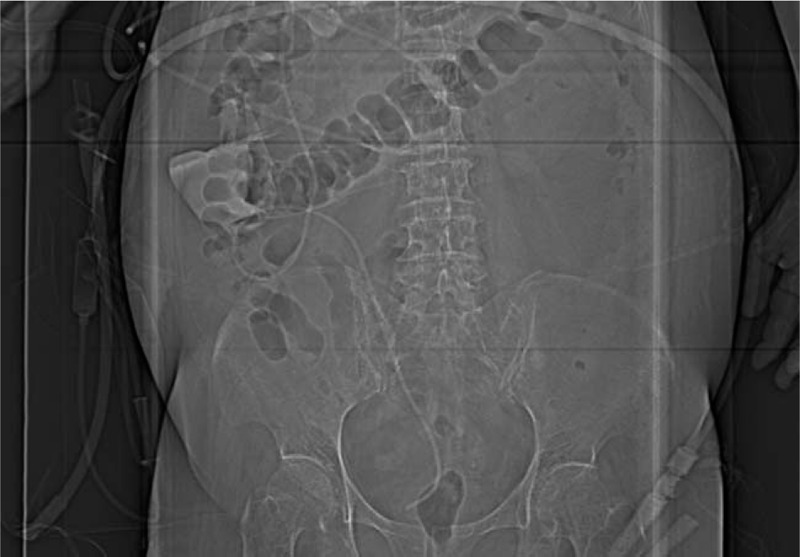
Postoperative head CT scan obtained during the second admission revealed the relief of ventricular enlargement and paraventricular edema. CT = computed tomography.

After we identified the absence of an underlying infection, laparoscopic cholecystectomy was performed under general anesthesia 7 days following drainage from the reservoir. Intraoperative exploration revealed the shunt tube to have been partially covered and severely curled by the omentum majus around the gallbladder (Fig. 5). We also observed elevated pelvic effusion but normal – albeit slow – cerebrospinal fluid outflow. The gallbladder was carefully and completely removed without bile leakage or bleeding. Antibiotics were administered regularly as suggested by the general surgery expert. The patient maintained wakefulness and could speak a few words with slowed speech. Her memory and cognitive ability were slightly impaired without a positive sign in the abdomen. A head CT scan showed the attenuation of ventricular enlargement and paraventricular edema (Fig. 6). No catheter or abdominal infection signs were observed during the subsequent 3 months of follow-up.

## 3 Discussion

Shunt malfunction refers to a partial or complete blockage of the shunt that partially or completely compromises its function. Although shunt malfunction has been attributed to multiple causes, including obstruction, infection, subdural hematoma, and hygroma,^[7]^ rare risk factors that result in shunt failure feature scant discussion in the literature. To the best of our knowledge, the present study is the first to report that chronic cholecystitis can induce shunt malfunction.

It is recommended that the initial valve operating pressure be set to between 30 and 70 mmH_2_O^[8]^; indeed, the symptoms of most patients improve when the final valve-operating pressure is set to 30 mmH_2_O.^[9]^ The most common problem among patients with shunts is symptomatic underdrainage caused by infections and obstructions soon following operation; their resolution requires the resetting of the valve operating pressure or adjustment of the distal catheter with revision surgery.^[10]^ However, our patient's condition did not improve even after the operating pressure was decreased to 0 mmH_2_O. After we confirmed the absence of an underlying infection, catheter revision surgery was excluded. Intraventricular pressure was approximately equal to the sum of the valve operating pressure and the intra-abdominal pressure (IAP).^[11]^ The mean normal IAP was 6.5 mm Hg (range 0.2–16.2 mm Hg).^[12]^ An IAP of over 10 mmHg is defined as intra-abdominal hypertension, which is categorized as either Grade 1, 2, or 3 according to the IAP^[13]^: 12–15/16-20/21-25 mm Hg, respectively. As abdominal infection is 1 of the most common factors that increases IAP,^[14]^ we speculatively attributed her symptoms to intra-abdominal hypertension induced by cholecystitis. The omentum can constrain the spread of intra-peritoneal infections by isolating the site of infection from adjacent healthy areas, and the movement of the omentum could have thereby distorted the original pathway of the shunt tube.^[15]^ The shunt tube having been partially covered and severely curled by the omentum majus surrounding the gallbladder, the displacement of the exudate of chronic cholecystitis and the omentum, and the imbalance between drainage and absorption could have thus contributed to the elevated pelvic effusion and IAP. The consequently lengthened outflow rate no longer met the requirements of the shunt, eventually causing it to malfunction.

Prior studies have attributed shunt malfunction to intra-abdominal hypertension. Chronic constipation is an important predisposing factor for distal malfunction in shunt-dependent hydrocephalus and should be suspected in case of a VPS malfunction.^[16]^ Martinez-Lage et al described 2 cases of reversible VPS failure related to severe constipation that reportedly resulted from increased intra-abdominal pressure linked to elevated ICP ^[17]^; this conclusion is supported by other reports.^[18,19]^ In addition, pregnancy and delivery have been implicated in intra-abdominal hypertension: an enlarged uterus and the blockage of venous outflow or forceful uterine contractions that occur during labor may purportedly cause shunt failure during gestation and delivery as well as raise IAP.^[19,20]^ Intra-abdominal hypertension induced by obesity is also considered a significant risk factor for increased ICP and pseudo-tumor cerebri. Obesity increases pleural pressure and cardiac filling pressure that, in turn, cause the obstruction of venous return from the brain. Laparoscopic surgery has also been implicated in the failure of cerebrospinal fluid shunts.^[20]^ Uzzo et al found rapid, sustained increases in ICP of greater than 12 mm Hg above baseline to a maximum pressure of 25 mm Hg.^[21]^ Other abdominal infection disease have further been associated with VPS malfunction in children with necrotizing enterocolitis and hydrocephalus.^[22]^

A series of cases concerning iNPH patients who underwent VP shunt surgery and suffered from acute cholecystitis indicates that not all instances of cholecystitis will cause a shunt malfunction.^[23–25]^ We speculate that the inflammation observed in such cases was confined to a constrained area due to the action of the omentum, leaving the distal end of the shunt tube uncontaminated. Shunt malfunction may occur only when the shunt tube is severely distorted or curled after being wrapped by the omentum. The success of laparoscopic surgery in such cases indicates the safety of this intervention. The following strategies have been advanced to prevent increases in ICP: the reduced use of abdominal pressure, intraoperative ICP monitoring/ventricular drainage, and distal shunt catheter clamping/externalization.^[26,27]^

In the present case, laparoscopic cholecystectomy was successfully performed without intraoperative ICP monitoring or shunt manipulation, and routine anesthetic monitoring appears to be safe. Care should be exercised during trocar placement to avoid inadvertent damage to the shunt; this cautionary measure also applies to the peritoneal portion of the catheter during laparoscopy.^[24]^ Furthermore, subcutaneous emphysema should not be neglected along the catheter tract during the peritoneal insufflation.^[28]^ The operation requires an experienced general surgeon to minimize the risk of postoperative abdominal and intracranial infection.^[26]^ Large cohorts and randomized control trials are warranted to identify the long-term clinical benefits.

## Acknowledgments

The authors would like to thank the patient's family for giving consent.

## Author contributions

**Conceptualization:** Yunhui Liu, Qi Yu.

**Data curation:** Chengjian Lou, Tianda Feng.

**Investigation:** Qi Yu.

**Methodology:** Tianda Feng.

**Supervision:** Yunhui Liu.

**Writing – original:** Qi Yu.

**Writing – review & editing:** Yunhui Liu.
